# Supplementary material for: Association between oral microbiome and seven types of cancers in East Asian population: a two-sample Mendelian randomization analysis
Source: Front Mol Biosci. 2023 Nov 21;10:1327893. doi: 10.3389/fmolb.2023.1327893 (PMC10702768; doi:10.3389/fmolb.2023.1327893)
Supplement: Supplementary file 1 [file DataSheet1.ZIP › Supplementary Materials/MR plots for tongue/tongue═╝/Pancreatic cancer/╥╚╧┘░⌐_╔╕╤í╜ß╣√/pheno.2799_to_pancreatic cancer_leave_one_out.pdf]

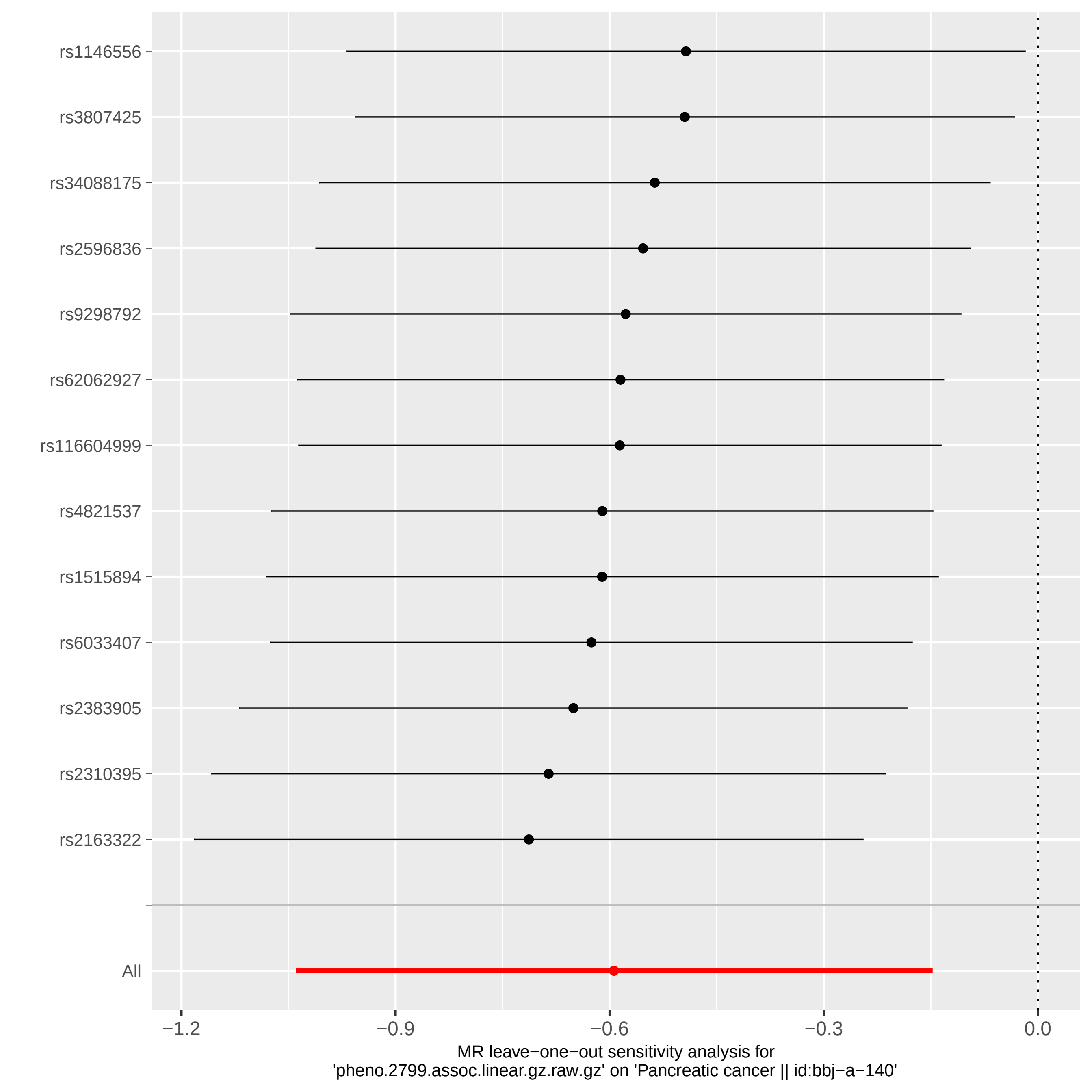
rs1146556
rs3807425
rs34088175
rs2596836
rs9298792
rs62062927
rs116604999
rs4821537
rs1515894
rs6033407
rs2383905
rs2310395
rs2163322
All
−1.2
−0.9
−0.6
−0.3
0.0
MR leave−one−out sensitivity analysis for
'pheno.2799.assoc.linear.gz.raw.gz' on 'Pancreatic cancer || id:bbj−a−140'
